# When Depression Masks Dementia: A Case of Dementia With Lewy Bodies Presenting With Suicidal Ideation

**DOI:** 10.1192/bjo.2026.11769

**Published:** 2026-06-30

**Authors:** Blerta Cenko, Yehudit Bauernfreund, Sarene Loganathan, Alastair Green, Begum Bingor

**Affiliations:** 1 North London Foundation Trust, London, United Kingdom; 2 Whittington health NHS trust, London, United Kingdom

## Abstract

**Aims:**

Dementia with Lewy bodies (DLB) is a neurodegenerative disorder that frequently presents with psychiatric symptoms, including depression, anxiety, psychosis, and suicidality, often before overt cognitive or motor deficits are apparent. Misdiagnosis as a primary psychiatric illness is common, and inappropriate exposure to antipsychotic medication can lead to severe adverse effects, including rigidity, hypersalivation, and profound functional decline. Early recognition of DLB in psychiatric settings is therefore critical. We present a case illustrating the diagnostic challenges of DLB initially presenting as late-onset psychotic depression with suicidal ideation.

**Methods:**

A 66-year-old man with no previous psychiatric history presented with a first episode of severe depression with psychotic features and repeated suicidal ideation. He was found by his wife holding a knife with reported intent to self-harm and was admitted to an inpatient ward under the Mental Health Act. These symptoms emerged after an injury that ended his employment. Prior to admission, he had been independent in activities of daily living and lived with his family.

During admission, he experienced rapid functional decline, including reduced verbal output, psychomotor retardation, and new-onset urinary incontinence. He was treated for psychotic depression with trials of risperidone and aripiprazole, both of which caused severe extrapyramidal side effects, including rigidity and hypersalivation, necessitating discontinuation. He was subsequently managed with low-dose sertraline, with partial symptom control.

**Results:**

Neurological examination demonstrated hypomimia, hypophonia, shuffling gait with reduced arm swing, resting tremor, bradykinesia, axial rigidity, postural instability, and ideomotor apraxia. Cognitive assessment was limited by poor engagement but revealed marked day-to-day fluctuations in attention and functional ability. Collateral history also suggested Rapid eye movement sleep behaviour symptoms.

The combination of fluctuating cognition, parkinsonism, pronounced antipsychotic sensitivity, and neuropsychiatric features met McKeith (2017) criteria for Probable DLB. Magnetic resonance and Dopamine Transporter scan supported this diagnosis. The patient was referred to the cognitive disorders’ clinic and neurology for further assessment, after initiation of rivastigmine. Clozapine was subsequently started to treat persistent psychotic symptoms, on neurology advice.

**Conclusion:**

This case demonstrates that DLB can present primarily as late-onset depression with psychotic features and suicidality, preceding clear cognitive or motor signs. Key clinical red flags include rapid functional decline, fluctuating cognition, parkinsonism, and severe sensitivity to antipsychotics. Early identification of these features is essential to prevent iatrogenic harm, guide safe pharmacological management, and enable timely referral to specialist services, including cognitive disorders clinics and neurology, for comprehensive assessment and long-term care planning.

